# Excellent effects and possible mechanisms of action of a new antibody–drug conjugate against EGFR-positive triple-negative breast cancer

**DOI:** 10.1186/s40779-021-00358-9

**Published:** 2021-12-09

**Authors:** Dan-Dan Zhou, Wei-Qi Bai, Xiao-Tian Zhai, Li-Ping Sun, Yong-Su Zhen, Zhuo-Rong Li, Qing-Fang Miao

**Affiliations:** 1 NHC Key Laboratory of Biotechnology of Antibiotics, Institute of Medicinal Biotechnology, Chinese Academy of Medical Sciences and Peking Union Medical College, No.1 Tiantan Xili, Beijing, 100050 China; 2 Department of Organic Chemistry, Institute of Medicinal Biotechnology, Chinese Academy of Medical Sciences and Peking Union Medical College, No.1 Tiantan Xili, Beijing, 100050 China

**Keywords:** Triple-negative breast cancer, Epidermal growth factor receptor, Antibody–drug conjugate, Targeted therapy, Antitumor effect

## Abstract

**Background:**

Triple-negative breast cancer (TNBC) is the most aggressive subtype and occurs in approximately 15–20% of diagnosed breast cancers. TNBC is characterized by its highly metastatic and recurrent features, as well as a lack of specific targets and targeted therapeutics. Epidermal growth factor receptor (EGFR) is highly expressed in a variety of tumors, especially in TNBC. LR004-VC-MMAE is a new EGFR-targeting antibody–drug conjugate produced by our laboratory. This study aimed to evaluate its antitumor activities against EGFR-positive TNBC and further studied its possible mechanism of antitumor action.

**Methods:**

LR004-VC-MMAE was prepared by coupling a cytotoxic payload (MMAE) to an anti-EGFR antibody (LR004) via a linker, and the drug-to-antibody ratio (DAR) was analyzed by HIC-HPLC. The gene expression of EGFR in a series of breast cancer cell lines was assessed using a publicly available microarray dataset (GSE41313) and Western blotting. MDA-MB-468 and MDA-MB-231 cells were treated with LR004-VC-MMAE (0, 0.0066, 0.066, 0.66, 6.6 nmol/L), and the inhibitory effects of LR004-VC-MMAE on cell proliferation were examined by CCK-8 and colony formation. The migration and invasion capacity of MDA-MB-468 and MDA-MB-231 cells were tested at different LR004-VC-MMAE concentrations (2.5 and 5 nmol/L) with wound healing and Transwell invasion assays. Flow cytometric analysis and tumorsphere-forming assays were used to detect the killing effects of LR004-VC-MMAE on cancer stem cells in MDA-MB-468 and MDA-MB-231 cells. The mouse xenograft models were also used to evaluate the antitumor efficacy of LR004-VC-MMAE in vivo. Briefly, BALB/c nude mice were subcutaneously inoculated with MDA-MB-468 or MDA-MB-231 cells. Then they were randomly divided into 4 groups (*n* = 6 per group) and treated with PBS, naked LR004 (10 mg/kg), LR004-VC-MMAE (10 mg/kg), or doxorubicin, respectively. Tumor sizes and the body weights of mice were measured every 4 days. The effects of LR004-VC-MMAE on apoptosis and cell cycle distribution were analyzed by flow cytometry. Western blotting was used to detect the effects of LR004-VC-MMAE on EGFR, ERK, MEK phosphorylation and tumor stemness marker gene expression.

**Results:**

LR004-VC-MMAE with a DAR of 4.02 were obtained. The expression of EGFR was found to be significantly higher in TNBC cells compared with non-TNBC cells (*P* < 0.01). LR004-VC-MMAE inhibited the proliferation of EGFR-positive TNBC cells, and the IC_50_ values of MDA-MB-468 and MDA-MB-231 cells treated with LR004-VC-MMAE for 72 h were (0.13 ± 0.02) nmol/L and (0.66 ± 0.06) nmol/L, respectively, which were significantly lower than that of cells treated with MMAE [(3.20 ± 0.60) nmol/L, *P* < 0.01, and (6.60 ± 0.50) nmol/L, *P* < 0.001]. LR004-VC-MMAE effectively inhibited migration and invasion of MDA-MB-468 and MDA-MB-231 cells. Moreover, LR004-VC-MMAE also killed tumor stem cells in EGFR-positive TNBC cells and impaired their tumorsphere-forming ability. In TNBC xenograft models, LR004-VC-MMAE at 10 mg/kg significantly suppressed tumor growth and achieved complete tumor regression on day 36. Surprisingly, tumor recurrence was not observed until the end of the experiment on day 52. In a mechanistic study, we found that LR004-VC-MMAE significantly induced cell apoptosis and cell cycle arrest at G_2_/M phase in MDA-MB-468 [(34 ± 5)% vs. (12 ± 2)%, *P* < 0.001] and MDA-MB-231 [(27 ± 4)% vs. (18 ± 3)%, *P* < 0.01] cells. LR004-VC-MMAE also inhibited the activation of EGFR signaling and the expression of cancer stemness marker genes such as Oct4, Sox2, KLF4 and EpCAM.

**Conclusions:**

LR004-VC-MMAE showed effective antitumor activity by inhibiting the activation of EGFR signaling and the expression of cancer stemness marker genes. It might be a promising therapeutic candidate and provides a potential therapeutic avenue for the treatment of EGFR-positive TNBC.

**Supplementary Information:**

The online version contains supplementary material available at 10.1186/s40779-021-00358-9.

## Background

Triple-negative breast cancer (TNBC), characterized by the absence or low expression of estrogen receptor (ER), progesterone receptor (PR), and human epidermal growth factor receptor 2 (HER2), is the most aggressive subtype of breast cancer [1]. TNBC is known for strong invasiveness, high relapse rates and poor overall survival and accounts for 15–20% of all breast cancer cases [2]. Due to lack of specific therapeutic targets, nonspecific treatments, such as surgery, conventional chemotherapy and radiotherapy have been the only therapeutic options for the last 2 decades. And clinical outcomes for TNBC unfortunately remain unsatisfactory. The median overall survival for TNBC metastatic patients is approximately 18 months, much shorter than that for HR-positive and HER2-enriched disease, where survival may exceed 5 years [3]. Now this situation is gradually changing with the development of targeted therapy, and a few of targeted therapeutics have been approved, such as Atezolizumab (an anti-PD-L1 antibody) for PD-L1 positive unresectable locally advanced or metastatic TNBC and Sacituzumab govitecan for some patients with metastatic TNBC [4]. Targeted therapy is bringing new hope for TNBC patients.

Epidermal growth factor receptor (EGFR) is a receptor tyrosine kinase (RTK) that belongs to the ErbB family, and its activation is closely related to cell growth and carcinogenesis [5]. EGFR overexpression was found in 45–70% of TNBC patients and was associated with poor prognosis [6], so EGFR may be a potential tumor target for TNBC therapy. Currently, there are two main types of EGFR inhibitors: anti-EGFR monoclonal antibodies (mAbs) and small molecule tyrosine kinase inhibitors (TKIs), which have been widely used in the treatment of highly expressed EGFR tumors, including non-small-cell lung cancer, colon cancer, and pancreatic cancer [7–9]. Unfortunately, most TNBC patients responded poorly or developed resistance to these drugs, possibly because quickly evolving mechanisms can either activate an alternative pathway or restore EGFR signaling, which drives proliferation and survival of cancer cells and limit the efficacy of EGFR inhibitors. No EGFR-targeted drugs have been currently approved for the treatment of TNBC [10–12]. Therefore, novel therapeutics that kill EGFR-expressing cancer cells by an action mechanism different from EGFR inhibitors by suppressing EGFR function may be a potential option for TNBC treatments.

Antibody–drug conjugates (ADCs) are targeted cancer therapeutics that are chemically synthesized by combining mAbs and cytotoxic payloads with a linker [13, 14]. As one of the fastest growing fields in cancer therapy, ADCs can utilize antibodies to deliver cytotoxic drugs directly to cancer cells and kill them, which reduces systemic exposure and toxicity [15]. Currently, eleven ADCs have been approved to treat cancer, but only one ADC (Sacituzumab govitecan-hziy), which targets trophoblast cell-surface antigen 2 (TROP-2), is used to treat TNBC [16]. Several EGFR-targeting ADCs (such as AVID-100, ABT-414 and IMGN289) have also entered clinical trials [17–19]. However, EGFR-targeted ADCs for TNBC therapy have not been reported. We previously reported that a novel ADC (LR004-VC-MMAE), which was composed of an anti-EGFR antibody (LR004) tethered to the cytotoxic drug monomethyl auristatin E (MMAE) via a chemical linker, showed potent antitumor effects against esophageal squamous cell carcinoma both in vitro and in vivo [20]. In this study, we further investigated the antitumor efficacy and possible molecular mechanism of LR004-VC-MMAE in EGFR-overexpressing TNBCs.

## Methods

### Antibodies and reagents

Primary antibodies against EGFR (4267S), phosphorylated EGFR (pEGFR) (3777 T), extracellular signal-regulated kinase (ERK) (4695S), phosphorylated ERK (pERK) (4370 T), mitogen-activated protein kinase kinase (MEK) (4694S), phosphorylated MEK (pMEK) (3958S), and β-actin (4970S) were purchased from Cell Signaling Technology (Boston, USA). Primary antibodies anti-CD24 conjugated with APC and anti-CD44 conjugated with PE were purchased from Biolegend (Beijing, China). Anti-CD133 antibody conjugated with FITC was obtained from Bioss (Beijing, China). The secondary antibodies conjugated to HRP were obtained from Santa Cruz Biotech (Beijing, China). Cell culture medium and FBS were purchased from Thermo Fisher Scientific (Waltham, MA). Cycloheximide and doxorubicin were acquired from Med Chem Express (New Jersey, USA). BCA protein assay kit and RIPA lysis buffer were obtained from Beyotime (Shanghai, China). CCK-8 assay kit and Annexin V-FITC apoptosis kit were purchased from Dojindo (Tokyo, Japan). Fibronectin and Matrigel were acquired from BD (USA). StemXVivo Tumor Sphere Media was obtained from R&D Systems (USA).

### Preparation of LR004-VC-MMAE

LR004-VC-MMAE was synthesized as described previously [20]. In brief, LR004 in sodium chloride buffer (containing 0.025 mmol/L borate, 1 mmol/L DTPA, pH = 8) was mixed with approximately threefold Tris(2-carboxyethyl) phosphine and stirred for 2 h at 37 °C under the protection of nitrogen. More than eightfold VC-MMAE was quickly dropped into the reaction system and incubated on ice both for 1 h, and a 20-fold excess of cysteine was added over the drug linker to terminate the reaction. Finally, the ADC products were eluted and purified by passing through equilibrated Sephadex G-25 and the eluate was concentrated by centrifugal ultrafiltration. The conjugate was filtered through a 2-μm filter under sterile conditions and used in this study. The drug-to-antibody ratio (DAR) was determined by HIC-HPLC analysis.

### EGFR expression analysis using bioinformatics

For EGFR gene expression analysis, a published dataset was downloaded from the Gene Expression Omnibus (GEO) repository (GSE41313). GraphPad Prism 5 was used to visualize the expression of EGFR genes. The Kaplan–Meier plotter (http://kmplot.com/analysis/) is a comprehensive online platform that can assess the effect of 54,675 genes on survival based on 10,293 cancer samples, including TNBC patients. The correlation between EGFR expression and survival in TNBC patients was analyzed with Kaplan–Meier plotter.

### Cell lines and cell culture

Breast cancer cell lines were used. MDA-MB-468 and BT-549 cell lines were obtained from the Chinese Academy of Sciences (Shanghai, China). MDA-MB-231 and MCF-7 cell lines were from the stocks maintained in our laboratory. MDA-MB-468, MDA-MB-231 and MCF-7 cells were maintained in Dulbecco’s modified Eagle’s medium (DMEM) supplemented with 10% FBS. BT-549 cells were cultured in RPMI-1640 medium supplemented with 10% FBS. All the cells were cultured in a humidified incubator at 37 °C under an atmosphere of 5% CO_2_.

### Cell viability assay by CCK-8 and colony formation assay

The viability of EGFR-positive TNBC cells was determined by using the CCK-8 assay kit according to the manufacturer’s protocol. In brief, MDA-MB-468 and MDA-MB-231 cells were seeded in 96-well plates (5000 cells/well) and incubated for 24 h. Next, the cells were treated with different concentrations of LR004-VC-MMAE (0, 0.0066, 0.066, 0.66 and 6.6 nmol/L) for 48 and 72 h. Then a mixture of 10 µl CCK-8 reaction solution and 90 µl complete culture medium was added to each well and incubated for 1 h. The absorbance was measured at 450 nm, and the experiments were repeated three times.

For colony formation assay, MDA-MB-468 or MDA-MB-231 cells were digested with trypsin and seeded in 6-well plates (2000 cells/well). The cells were treated with different concentrations of LR004-VC-MMAE (0, 0.5 and 1 nmol/L) and cultured for 1 week. After discarding the culture supernatant and washing the cells with PBS, 4% paraformaldehyde was added to fix the cells. After 25 min, the cells were washed, stained with crystal violet solution for 30 min, washed again with PBS and dried at room temperature. The number of colonies containing more than 50 cells in each well was counted under a microscope.

### In vitro cell migration and invasion abilities evaluated by wound healing and transwell assays

For cell migration assays, MDA-MB-468 or MDA-MB-231 cells were seeded in 6-well plates and cultured in an incubator at 37 °C in 5% CO_2_ until confluent. Then, the cells were gently scratched with a yellow pipette tip across the center of the well to create a wound. The cells were washed with PBS to remove dead cells and debris. The remaining cells were treated with 2.5 or 5 nmol/L LR004-VC-MMAE and then cultured in DMEM supplemented with 0.4% FBS for 24 h to allow wound healing. Images were captured at 0 h and 24 h after the wound was created with a microscope and recorded as D_0_ and D_24_, respectively. This assay was repeated three times, and the wound field was measured. The cell migration rate (%) = (D_0_ − D_24_)/D_0_ × 100%.

In cell invasion assays, Transwell chambers with filter membranes of 8-μm pore size were used. The chamber was pre-coated with 30 μl fibronectin (10 μg/ml) on the lower surface, and a polycarbonate filter was coated with 10 mg Matrigel. Then, the chamber was inserted in 24-well culture plates filled with complete medium. MDA-MB-468 or MDA-MB-231 cells were pretreated with different concentrations of LR004-VC-MMAE (2.5 and 5 nmol/L) for 48 h and seeded into the upper chamber (1 × 10^5^ cells/well in 0.4% FBS in DMEM medium). The cells that did not invade into Matrigel were gently removed using a cotton swab after 24 h. The invaded cells were fixed with 4% paraformaldehyde, stained with 0.25% crystal violet staining solution and counted under an inverted microscope.

### Cancer stem cell (CSC) marker expression analysis using flow cytometry

MDA-MB-468 or MDA-MB-231 cells were digested with trypsin, seeded in 6-well plates and cultured for 24 h. Subsequently, the cells were treated with 5 nmol/L LR004-VC-MMAE for 48 h. Then, the cells were collected, washed and incubated for 2 h with fluorochrome-conjugated antibodies (anti-CD24 conjugated with APC, anti-CD44 conjugated with PE and anti-CD133 conjugated with FITC). Cells were washed with cold PBS and analyzed by flow cytometry. The data were analyzed using ACEA NovoExpress software.

### CSC killing effects assessed by tumorsphere formation assay

MDA-MB-468 or MDA-MB-231 cells were digested with trypsin, counted and resuspended in complete StemXVivo Tumor Sphere Media. Then, the cells were seeded into a special 96-well plate (2000 cells/well) and treated with different concentrations of LR004-VC-MMAE (0, 0.5 and 1 nmol/L). Next, the cells were cultured in an incubator with 5% CO_2_ at 37 °C. Tumorsphere numbers were counted and photographed after 2 weeks of incubation.

### Apoptosis and cell cycle analysis by flow cytometry

MDA-MB-468 or MDA-MB-231 cells were seeded in a 6-well plate and treated with 10 or 50 μmol/L LR004-VC-MMAE for 48 h. Then they were harvested with trypsin, and washed with PBS. For apoptosis analysis, the cells were resuspended in 100 μl binding buffer and stained with 5 μl Annexin V and 5 μl propidium iodide (PI) for 15 min at room temperature in the dark. Next, mixed with 400 μl binding buffer and analyzed using an ACEA NovoExpress flow cytometer within an hour. For cell cycle analysis, the cells were fixed with 75% cold alcohol at 4 °C overnight. Next, resuspended in 0.5 ml PI/RNase staining buffer for 30 min in the dark at room temperature and analyzed using an ACEA NovoExpress flow cytometer. Cell cycle distribution was calculated with ACEA NovoExpress software.

### Western blotting for EGFR protein stability analysis

To assess EGFR protein stability, MDA-MB-231 cells were treated with a protein synthesis inhibitor Cycloheximide (20 mmol/L) for indicated durations of time after treatment with LR004-VC-MMAE for 2 h. Then cells were collected and lysed with RIPA lysis buffer. The protein was extracted and its concentration was determined using a BCA protein assay kit. The protein samples were separated on SDS-PAGE and transferred to polyvinylidene difluoride membranes for immunoblotting. The immunolabeled proteins were detected by an ECL detection system.

### In vivo antitumor efficacy

Twenty-four female athymic BALB/c nude mice (6-week-old) were purchased from Beijing HFK Bioscience Co., Ltd. [Beijing, China, SCXK(Beijing)2019-0008]. All animals were maintained in the animal facilities at the Institute of Medicinal Biotechnology under specific pathogen-free (SPF) conditions. All animal studies were approved by the Ethics Committee of the Institute of Medicinal Biotechnology, Chinese Academy of Medical Sciences (No. IMB-20200920D601). The animal experiments were performed in accordance with the ARRIVE guidelines [21].

For the tumor xenograft model, 5.0 × 10^6^ MDA-MB-468 cells or 3.0 × 10^6^ MDA-MB-231 cells were injected subcutaneously into the right flank of each 6‑week‑old female BALB/c nude mice. when the tumor volumes reached approximately 100 mm^3^ (approximately 8 days), the 24 mice were randomly divided into 4 groups (6 mice per group): control group, LR004 antibody group, LR004-VC-MMAE group, and doxorubicin (positive control) group, which were treated with PBS or different drugs (LR004 antibody 10 mg/kg, LR004-VC-MMAE 10 mg/kg, doxorubicin 2 mg/kg) every 5 days for 4 times through *i.v.* injections. Tumor growth was monitored every 4 days by measuring tumor length (L) and width (W) using Vernier calipers. The body weights of all mice were also measured. Tumor volume (TV) was calculated according to the following formula: TV = 0.5 × L × W^2^.

### Statistical analysis

Data analysis was performed with GraphPad Prism 5 software, and the data are presented as the mean ± standard deviation (SD). The different groups were analyzed using two-way ANOVA followed by Tukey’s test for pairwise comparison using GraphPad Prism 5. All experiments were repeated more than 3 times. *P* values of < 0.05 were considered statistically significant.

## Results

### Structure and composition of LR004-VC-MMAE

Building on our previous study, we first synthesized an EGFR-targeted ADC (LR004-VC-MMAE), which consists of an anti-EGFR antibody (LR004), a valine-citrulline (VC) dipeptide linker and a cytotoxic payload (MMAE) (Additional file 1: Fig. S1). DAR was determined by HIC-HPLC analysis. The HIC-HPLC spectrum of LR004-VC-MMAE displays 5 major peaks, corresponding to 0, 2, 4, 6, and 8 drugs per antibody. The average DAR of 4.02 was achieved according to the HIC-HPLC analysis (Additional file 1: Fig. S1).

### EGFR is highly expressed and associated with poor survival in TNBC patients

**Fig. 1:**
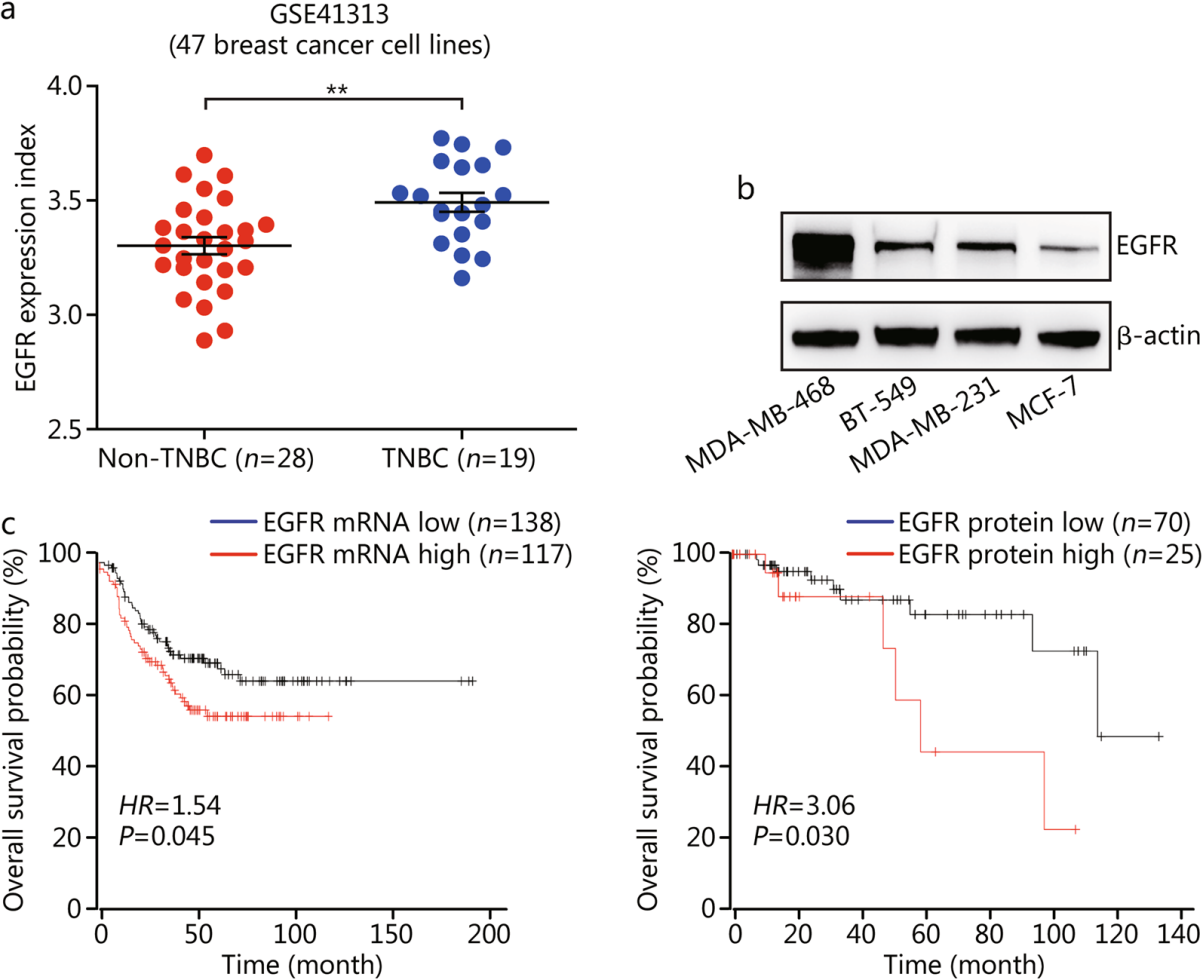
EGFR expression in TNBC cell lines and TNBC patients. **a** Quantitative analysis of EGFR transcript expression levels in non-TNBC and TNBC cell lines using a publicly available microarray dataset (GSE41313). Statistical significance was determined by Student’s *t*-test; ^**^P < 0.01. **b** EGFR protein expression in the indicated breast cancer cells. **c** Kaplan–Meier plot of overall survival of patients with TNBC. Patients were divided into 2 groups, high EGFR expression vs. low EGFR expression according to EGFR mRNA expression level (left) and protein expression level (right). EGFR epidermal growth factor receptor, TNBC triple-negative breast cancer

First, we assessed the gene expression of EGFR in a series of breast cancer cell lines using a publicly available microarray dataset (GSE41313). The results showed that the expression of EGFR transcript was significantly higher in TNBC cells than in non-TNBC cells (*P* < 0.01, Fig. 1a). Similarly, Western blotting results showed that EGFR protein levels were higher in TNBC cell lines (BT-549, MDA-MB-468 and MDA-MB-231) than in the non-TNBC MCF-7 cell line (Fig. 1b). In addition, Kaplan–Meier survival analysis revealed that higher expression of EGFR at the mRNA and protein levels were associated with a shorter overall survival in TNBC patients (Fig. 1c). These results demonstrated that EGFR was overexpressed in TNBC patients, which closely correlated with their poor survival.

### LR004-VC-MMAE reduces the viability of EGFR-positive TNBC cells

**Fig. 2:**
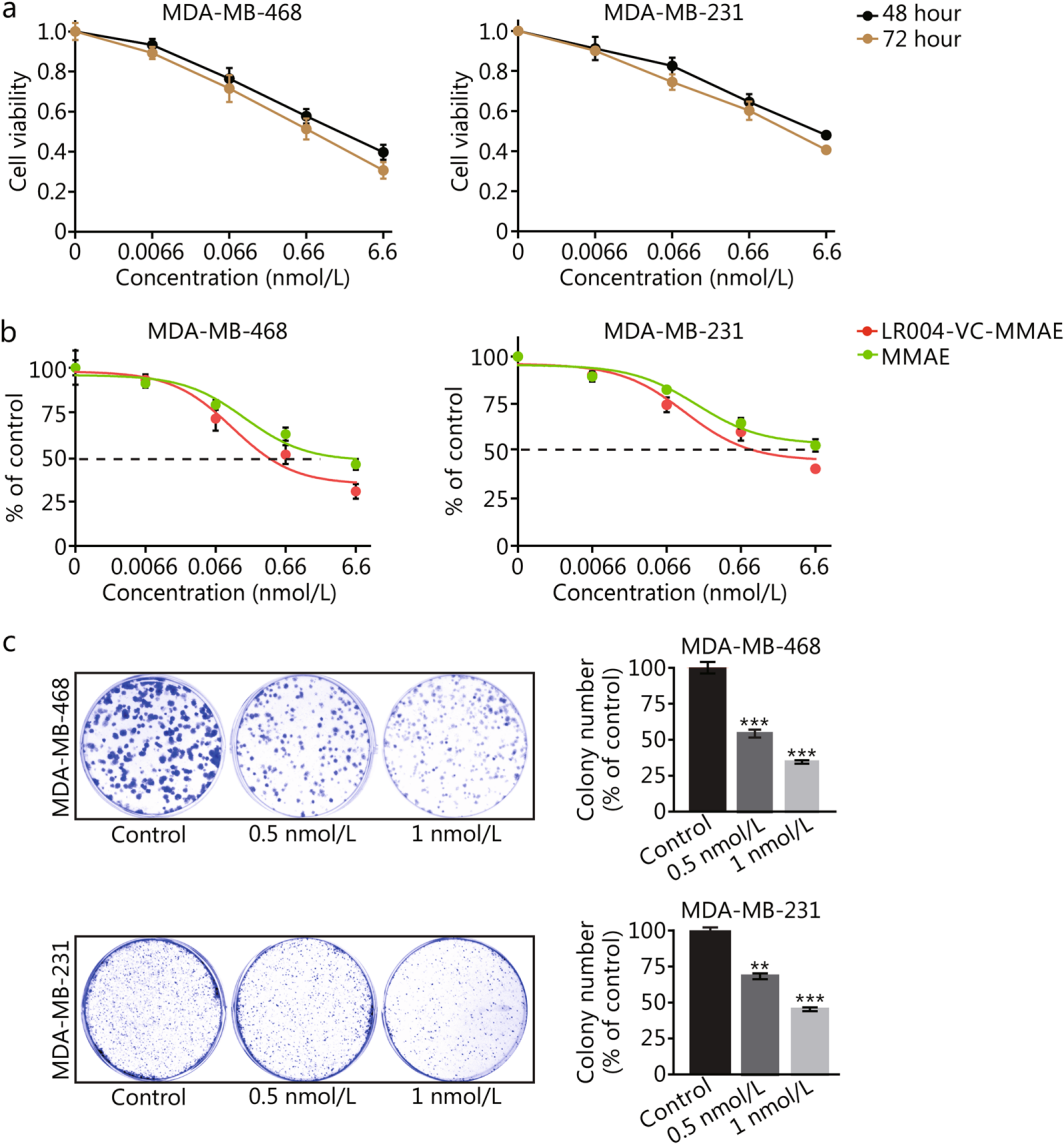
Effects of LR004-VC-MMAE on proliferation and colony formation of TNBC cells. **a** Effect of LR004-VC-MMAE on MDA-MB-468 and MDA-MB-231 cells viability. CCK-8 assays were used to assess the viability of cells treated with LR004-VC-MMAE for 48 or 72 h. **b** IC_50_ values of MDA-MB-468 and MDA-MB-231 cells. Cells were treated with LR004-VC-MMAE or MMAE for 72 h. The data are presented as the mean ± SD. **c** Colony forming ability of MDA-MB-468 and MDA-MB-231 cells. Representative images and colony counts (% of control) are shown. Data are presented as the mean ± SD. **P < 0.01, ***P < 0.001 vs. control. TNBC triple-negative breast cancer

According to the results in Fig. 1b, we used EGFR-overexpressing TNBC cell lines MDA-MB-468 and MDA-MB-231 to evaluate the effects of LR004-VC-MMAE on cell proliferation. The CCK-8 assay results showed that LR004-VC-MMAE inhibited the growth of MDA-MB-468 and MDA-MB-231 (Fig. 2a) cells in a dose- and time-dependent manner. The IC_50_ value of MDA-MB-468 cells treated with LR004-VC-MMAE for 72 h was (0.13 ± 0.02) nmol/L, which was significantly lower than that of cells treated with MMAE [(3.20 ± 0.60) nmol/L, *P* < 0.01] (Fig. 2b). And the IC_50_ value of MDA-MB-231 cells treated with LR004-VC-MMAE for 72 h was (0.66 ± 0.06) nmol/L, which was also significantly lower than that of cells treated with MMAE [(6.60 ± 0.50) nmol/L, *P* < 0.001] (Fig. 2b). Colony formation assays confirmed that the colony forming ability was significantly reduced after treatment with LR004-VC-MMAE in a dose-dependent manner. Compared with the control sample, the colony formation rate was less than (34 ± 3)% for MDA-MB-468 cells (*P* < 0.001, Fig. 2c) or (45 ± 1)% for MDA-MB-231 cells (*P* < 0.001, Fig. 2c) at a concentration of 1 nmol/L. These results indicated that LR004-VC-MMAE had excellent anti-proliferative activity in EGFR-positive TNBC cells.

### LR004-VC-MMAE inhibits the migration and invasion of EGFR-positive TNBC cells

**Fig. 3:**
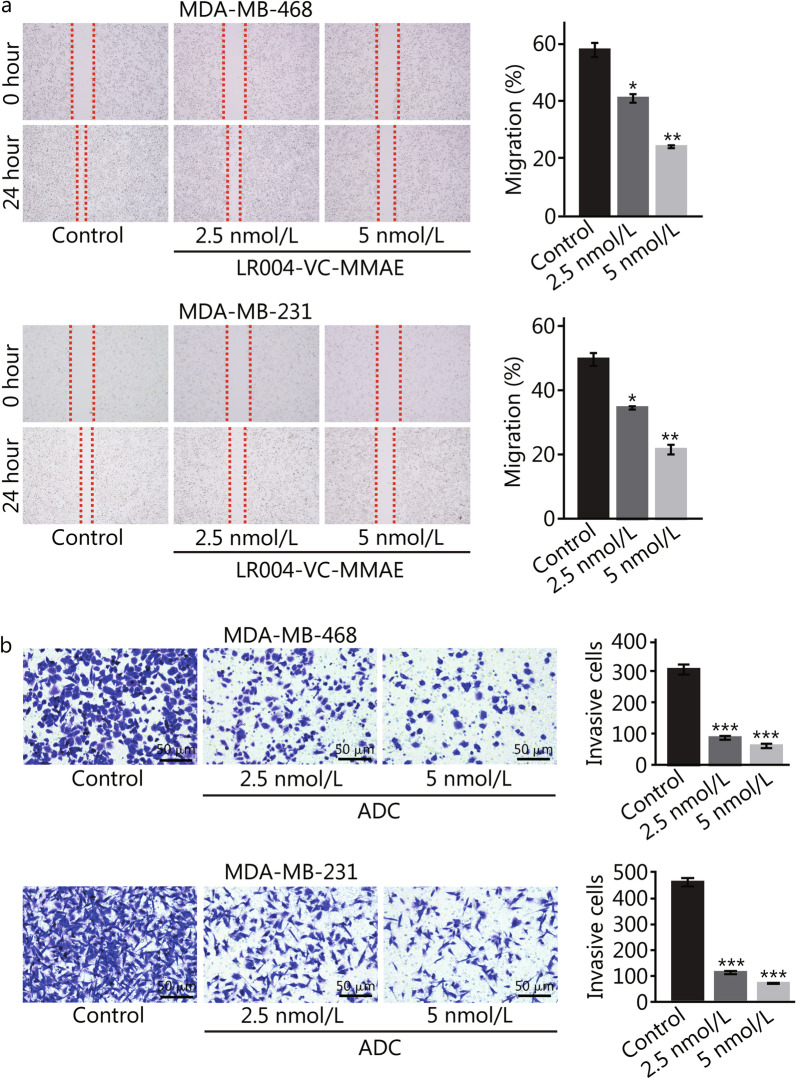
Effects of LR004-VC-MMAE on migration and invasion of TNBC cells. **a** Migration images of MDA-MB-468 and MDA-MB-231 cells. Magnification, 200×. Cells were treated with 2.5 or 5 nmol/L LR004-VC-MMAE for 24 h. **b** Invasion images of MDA-MB-468 and MDA-MB-231 cells were stained by coomassie brilliant blue. Magnification, 200×. Cells were treated with 2.5 or 5 nmol/L LR004-VC-MMAE for 24 h. Data represent the mean ± SD. *P < 0.05, **P < 0.01, ***P < 0.001 vs. control. TNBC triple-negative breast cancer

Following experiments were performed to further examine anti-migration and invasion effects of LR004-VC-MMAE on EGFR-positive TNBC cells. First, wound healing assays showed that cell migration was strongly inhibited in a dose-dependent manner after LR004-VC-MMAE treatment for 24 h. For MDA-MB-468 and MDA-MB-231 cells, the migration rates were (24 ± 1)% and (23 ± 3)%, respectively, after treatment with 5 nmol/L LR004-VC-MMAE, which were significantly lower than those of the control samples [(60 ± 3)% and (51 ± 2)%, *P* < 0.01] (Fig. 3a); in the Transwell invasion assay, compared to the control group, treatment with 2.5 nmol/L and 5 nmol/L LR004-VC-MMAE showed invasion inhibition rates of (70 ± 4)% and (82 ± 3)% for MDA-MB-468 cells (*P* < 0.001, Fig. 3b) and (73 ± 2)% and (81 ± 3)% for MDA-MB-231 cells (*P* < 0.001, Fig. 3b), respectively. These data suggested that LR004-VC-MMAE inhibited the migration and invasion ability of EGFR-positive TNBC cells.

### LR004-VC-MMAE reduces the fraction of CSCs and tumorsphere formation in EGFR-positive TNBC cells

**Fig. 4:**
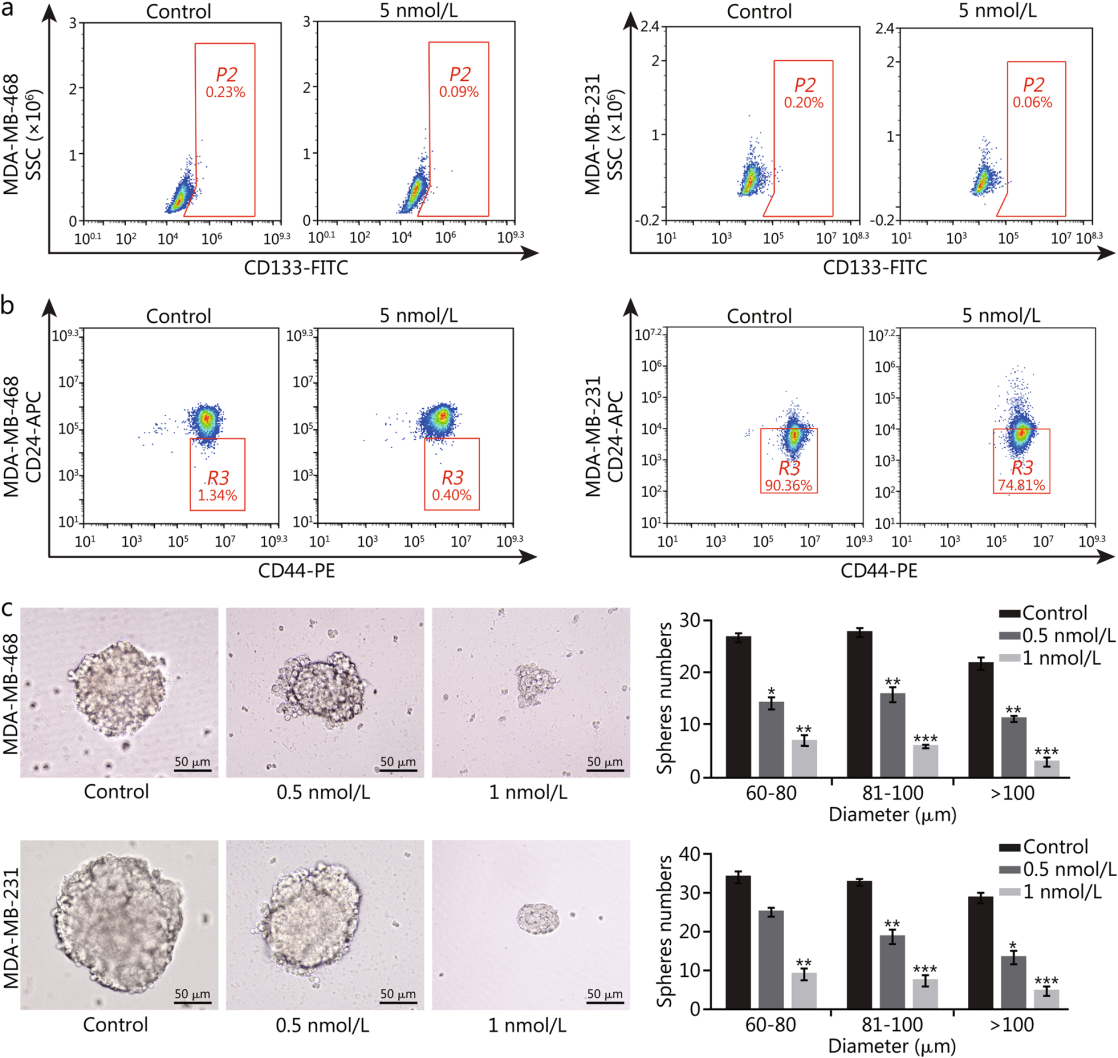
Inhibitory effects of LR004-VC-MMAE on CSCs in TNBC cells. **a** Proportions of CD133^+^ CSCs subpopulation in MDA-MB-468 and MDA-MB-231 cells. The cells were analyzed by FACS after treatment with 5 nmol/L LR004-VC-MMAE for 48 h. **b** Proportions of CD44^+^/CD24^−/low^ CSCs subpopulation in MDA-MB-468 and MDA-MB-231 cells. The cells were analyzed by FACS after treatment with 5 nmol/L LR004-VC-MMAE for 48 h. **c** Tumorspheres formed by MDA-MB-468 and MDA-MB-231 cells. The cells were treated with the indicated concentrations of LR004-VC-MMAE. Representative images of tumorspheres and the enumeration of tumorspheres are shown. *P < 0.05, **P < 0.01, ***P < 0.001 vs. control. SSC side scatter, CSC cancer stem cell, TNBC triple-negative breast cancer

Breast CSCs express a series of surface markers, such as CD44^+^, CD24^−^, CD133^+^, and ALDH^+^. To determine the effect of LR004-VC-MMAE on CSCs, we first investigated the fraction of CSCs by assessing the expression of CSC markers CD44, CD24 and CD133 in cancer cells by flow cytometry. The results indicated that the proportions of CD133^+^ and CD44^+^/CD24^−/low^ CSCs were reduced in MDA-MB-468 cells after LR004-VC-MMAE treatment. As shown in Fig. 4a and b, the proportion of CD133^+^ CSCs decreased from (0.230 ± 0.014)% to (0.090 ± 0.007)% (*P* < 0.001), and CD44^+^/CD24^−/low^ CSCs decreased from (1.340 ± 0.122)% to (0.400 ± 0.049)% (*P* < 0.001). Similar results were obtained in MDA-MB-231 cells, the proportion of CD133^+^ CSCs decreased from (0.200 ± 0.032)% to (0.060 ± 0.005)% (*P* < 0.01, Fig. 4a), and CD44^+^/CD24^−/low^ CSCs reduced from (90.360 ± 4.278)% to (74.810 ± 2.866)% (*P* < 0.05, Fig. 4b). Then, we performed a tumorsphere formation assay to evaluate the killing effect of LR004-VC-MMAE on CSCs. As presented in Fig. 4c, LR004-VC-MMAE treatment significantly reduced the number and size of tumorspheres in both MDA-MB-468 and MDA-MB-231 cells in a concentration-dependent manner. In particular, compared with the control, the number of tumorspheres larger than 100 μm in diameter in MDA-MB-468 and MDA-MB-231 cells treated with 1 nmol/L LR004-VC-MMAE decreased by (85.0 ± 2.9)% (*P* < 0.001, Fig. 4c) and (82.0 ± 3.6)% (*P* < 0.001, Fig. 4c), respectively. All these results indicated that LR004-VC-MMAE had a significant cytotoxicity on tumor stem cells.

### LR004-VC-MMAE inhibits EGFR-positive TNBC growth in xenograft models

**Fig. 5:**
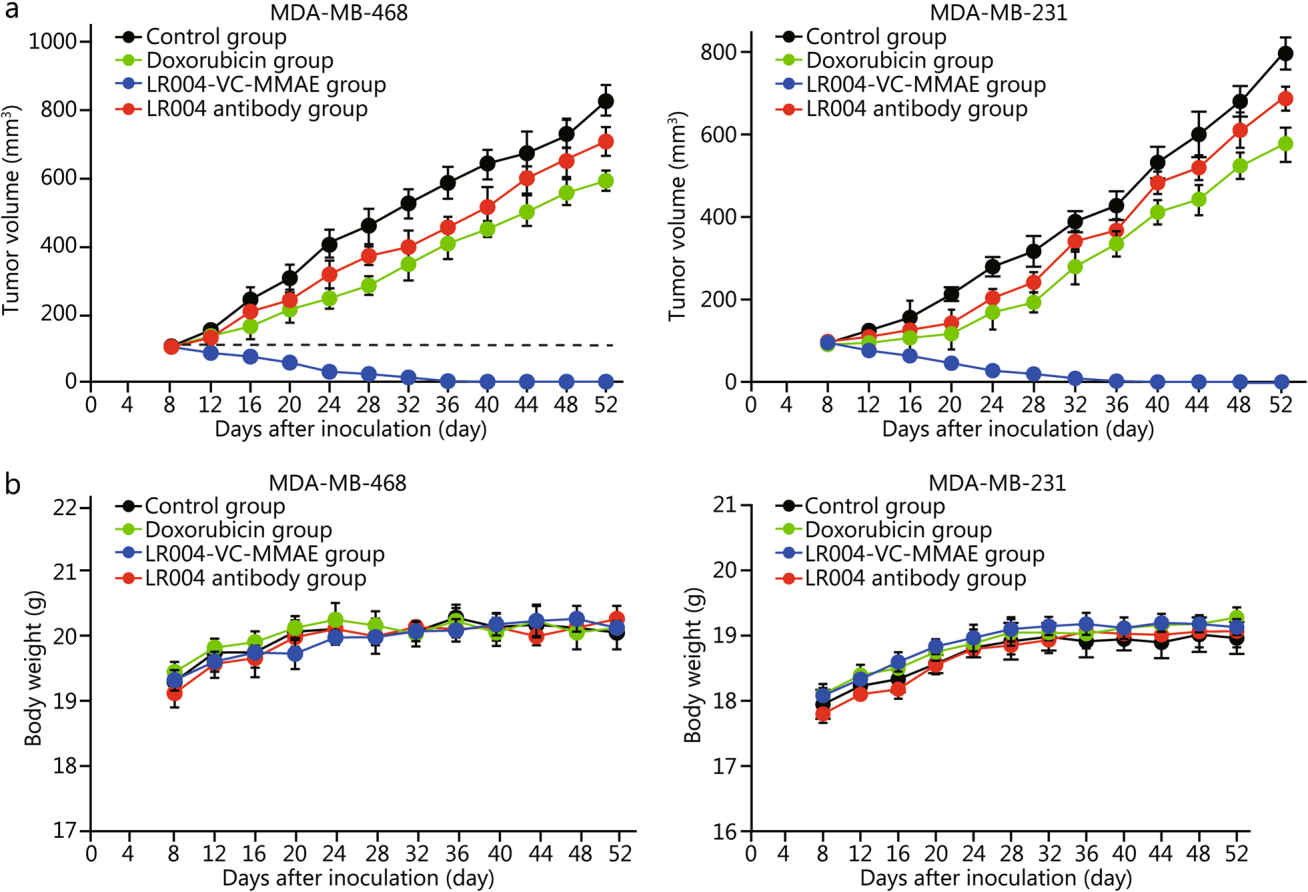
Therapeutic efficacy of LR004-VC-MMAE against TNBC in vivo. **a** Growth curves of MDA-MB-468 and MDA-MB-231 cell-derived xenograft tumors. Mice were inoculated with MDA-MB-468 or MDA-MB-231 cells and treated with PBS (control), naked antibody LR004 (10 mg/kg), doxorubicin (positive control, 2 mg/kg) or LR004-VC-MMAE (10 mg/kg) every 4 days via the tail vein for a total of four injections (*n* = 6). **b** Body weight curves of mice in (**a**)

Next, we evaluated the antitumor activities of LR004-VC-MMAE in subcutaneous TNBC xenograft models. On day 52 post-tumor implantation, the average tumor volumes in the control group, LR004 antibody group, and doxorubicin group were (820.5 ± 75.7) mm^3^, (718.5 ± 65.7) mm^3^, and (636.6 ± 45.6) mm^3^, respectively (Fig. 5a). Compared with the control group, the tumor inhibition rate of LR004 antibody group was (12.40 ± 0.92)% (*P* = 0.34), suggesting that the naked antibody LR004, as a single agent, had marginal antitumor activity in the MDA-MB-468 xenograft model. However, in the LR004-VC-MMAE group, tumors diminished in size after treatment, and eventually, all became undetectable by day 36. Moreover, tumor recurrence was not observed until the end of the experiment on day 52 (Fig. 5a). Similarly, the naked antibody LR004 had minimal antitumor activity in MDA-MB-231 xenograft model [tumor inhibition rate was (10.20 ± 1.04)% on day 52, *P* = 0.23]. Significant tumor regression was observed in LR004-VC-MMAE group, and tumors disappeared on day 36 (Fig. 5a). In addition, there was no significant weight loss in any group (Fig. 5b), indicating that there was no systemic toxicity of ADC treatment.

### LR004-VC-MMAE induces apoptosis and cell cycle arrest

**Fig. 6:**
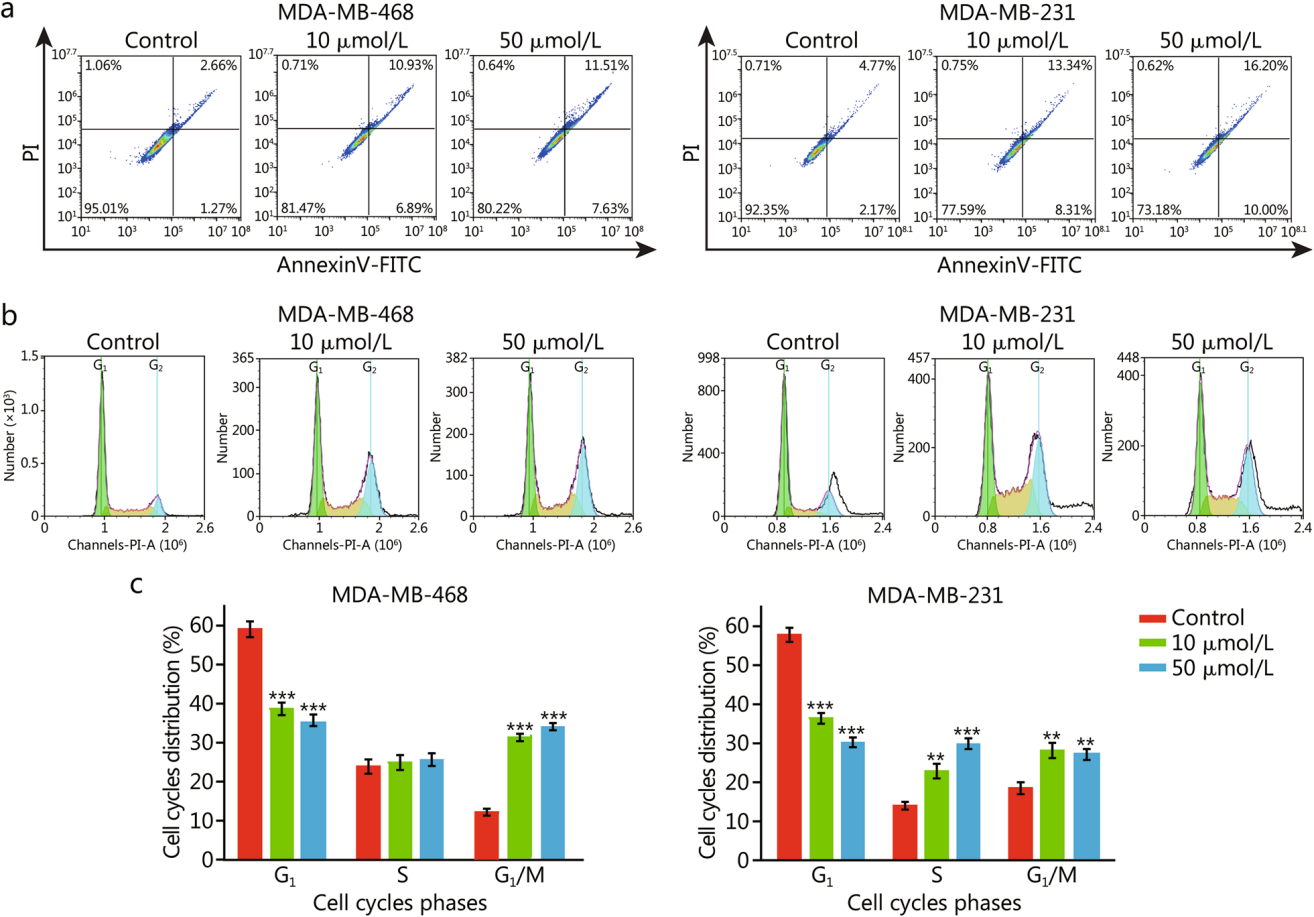
Effects of LR004-VC-MMAE on cell apoptosis and cell cycle phase distribution in TNBC cells. **a** Apoptosis analysis of MDA-MB-468 and MDA-MB-231 cells. Cell apoptosis was assessed by FACS after treatment with 10 or 50 μmol/L LR004-VC-MMAE for 48 h. **b** Cell cycle phase analysis in MDA-MB-468 and MDA-MB-231 cells. Cells were treated with 10 or 50 μmol/L LR004-VC-MMAE for 48 h, stained with propidium iodide (PI), and the cell cycle phase distribution was analyzed by FACS. **c** Quantitative analysis of the cell cycle phase distribution in MDA-MB-468 and MDA-MB-231 cells. The experiments were performed in triplicate. Data are presented as mean ± SD. **P < 0.01, ***P < 0.001 vs. control

Apoptosis and cell cycle distribution were analyzed to investigate the potential mechanism of tumor suppression by flow cytometry. The data showed that LR004-VC-MMAE significantly increased early apoptotic and late apoptotic cell populations in a concentration-dependent manner. For MDA-MB-468 cells, the apoptotic cell ratio was significantly increased from (3.93 ± 0.56)% of the control group to (19.14 ± 0.71)% (*P* < 0.01) when treated with 50 μmol/L LR004-VC-MMAE (Fig. 6a). Similar results were obtained in MDA-MB-231 cells, and the apoptosis rate was (26.20 ± 1.24)%, which was higher than that of the control group [(6.94 ± 0.63)%,* P* < 0.01] (Fig. 6a). In cell cycle distribution analysis, the percentage of MDA-MB-468 cells in G_1_ phase decreased from (59 ± 4)% in control cells to (35 ± 3)% (*P* < 0.001) after 48 h treatment with 50 μmol/L LR004-VC-MMAE, and the percentage of cells in G_2_/M phase significantly increased from (12 ± 2)% in control cells to (34 ± 5)% (*P* < 0.001, Fig. 6b and c). Similarly, MDA-MB-231 cells treated with 50 μmol/L LR004-VC-MMAE showed marked accumulation not only in G_2_/M phase [(27 ± 4)% vs. (18 ± 3)%, *P* < 0.01], but also in S phase [(30 ± 3)% vs. (14 ± 2)%, *P* < 0.001] compared with the control (Fig. 6b and c). Taken together, these results demonstrated that LR004-VC-MMAE induced apoptosis and cell cycle arrest at G_2_/M phase in MDA-MB-468 and MDA-MB-231 cells.

### LR004-VC-MMAE inhibits EGFR activation and downregulates stemness marker genes

**Fig. 7:**
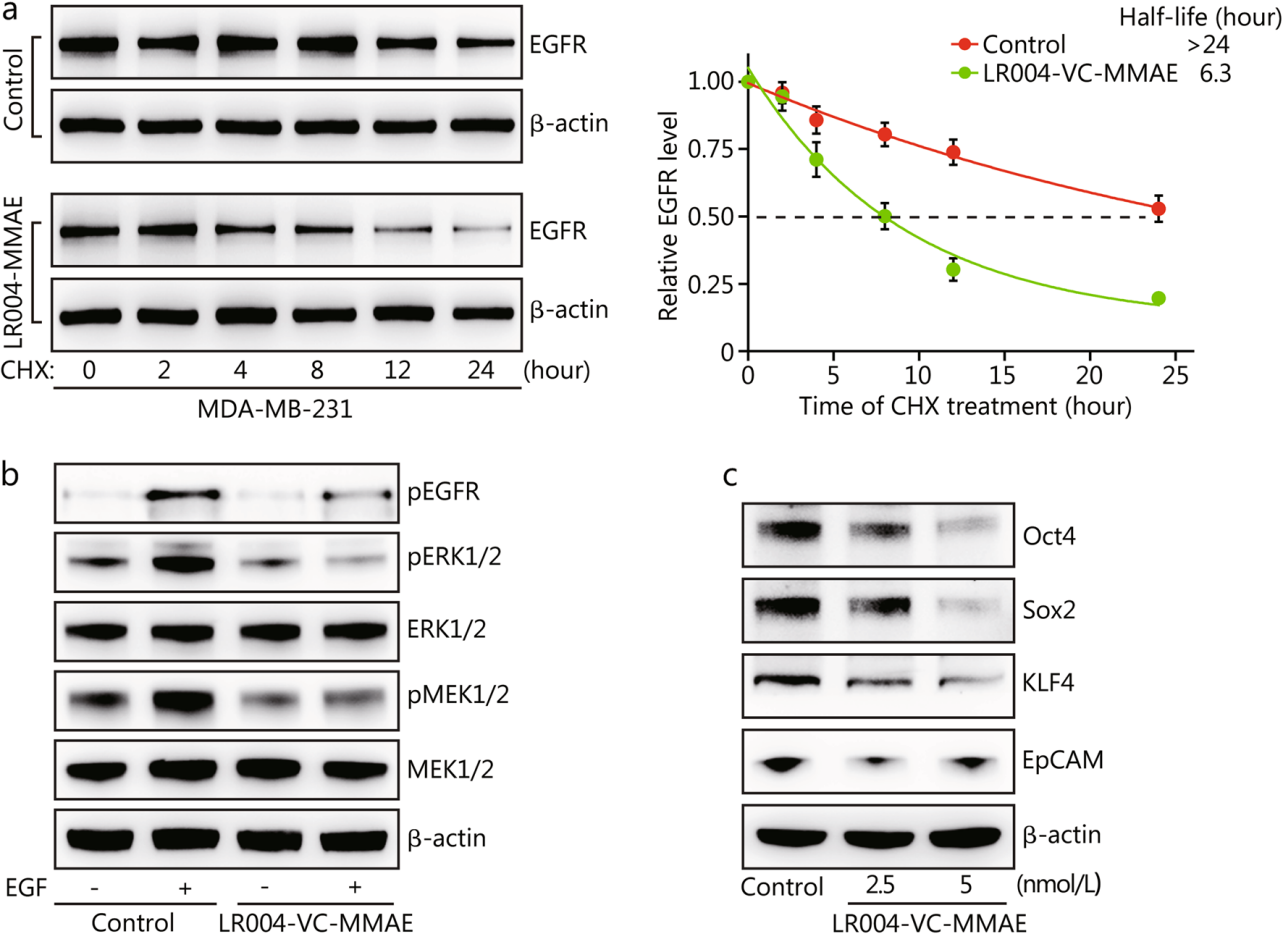
Effects of LR004-VC-MMAE on EGFR, EGFR downstream signal and the expression of tumor stemness marker genes. **a** Stability of EGFR protein in MDA-MB-231 cells. The cells pretreated with 2.5 nmol/L LR004-VC-MMAE or PBS (as a control) were incubated with cycloheximide (CHX) (20 mmol/L) for indicated durations. The EGFR protein levels were evaluated by immunoblotting. Representative images (left panel) and protein degradation curves (right panel) are shown. **b** Analysis of EGFR downstream signaling. Serum-starved MDA-MB-231 cells were treated with 2.5 nmol/L LR004-VC-MMAE or PBS for 24 h, followed by EGF (100 ng/ml) stimulation for 30 min. The indicated proteins and their phosphorylation status were evaluated by immunoblotting. **c** Expression of stem cell maker genes. MDA-MB-231 cells were treated with PBS or LR004-VC-MMAE (2.5 or 5 nmol/L) for 24 h. The indicated proteins were detected by immunoblotting

Subsequently, we explored the effect of LR004-VC-MMAE on the half-life of EGFR protein and found that EGFR half-life was much shorter in LR004-VC-MMAE-treated cells (6.3 h) than in the control cells (> 24 h, *P* < 0.001) (Fig. 7a). Next, cells were stimulated with EGF to activate the EGFR signaling pathway after preincubation with LR004-VC-MMAE for 24 h. Surprisingly, we found that EGF stimulation could not induce phosphorylation of EGFR or its downstream signaling molecules MEK or ERK1/2 (Fig. 7b). These data demonstrated that LR004-VC-MMAE not only promoted EGFR degradation but also repressed the activation of its downstream signals. Furthermore, LR004-VC-MMAE treatment also significantly reduced the expression of various cancer stemness marker genes, including Oct4, Sox2, KLF4 and EpCAM (Fig. 7c). These genes are important for the maintenance of cancer stemness and are responsible for tumor proliferation, migration, metastasis and relapse. The results suggest that downregulation of EGFR signaling by LR004-VC-MMAE may occur, at least in part, by inhibiting activation and expression of EGFR. Inhibition of the EGFR signaling pathway and the expression of cancer stemness marker genes may partially account for the antitumor effects of this compound.

## Discussion

TNBC is the most aggressive and challenging breast cancer subtype because of a high risk of recurrence, an earlier age of onset and a lack of recognized molecular targets for therapy [22]. There are fewer options available for treating TNBC, since both hormonal therapies and HER2 targeted therapies (such as Herceptin) are ineffective. While chemotherapy remains the mainstay of standard therapy, several novel treatments have been developed during the past few years, such as targeted therapy (poly ADP-ribose polymerase inhibitors and ADCs) and immunotherapy (programmed cell death-1 inhibitors, programmed cell death-ligand 1 inhibitors), which have significantly improved the prognosis of patients with TNBC [1, 2]. Among them, ADCs are one of the most promising classes of therapeutics. Currently, several ADCs have entered clinical trials for the treatment of TNBC, such as ladiratuzumab vedotin (SGN-LIV1a, targeting the zinc transporter LIV-1) [23] and U3-1402 (targeting HER3) [24]. Notably, sacituzumab govitecan (IMMU-132) was approved by the FDA in April 2020 for adult patients with metastatic TNBC who received at least two prior therapies for metastatic disease. This is the first and only ADC approved by the US FDA specifically for the treatment of patients with metastatic TNBC [25]. IMMU-132 is made from a humanized anti-Trop-2 mAb (hRS7) conjugated with the active metabolite of irinotecan (SN-38). In a single-arm phase I/II study, IMMU-132 was able to reduce tumor size in approximately one-third of the participants and triggered responses lasting a median of 7.7 months. In the treatment of TNBC, although IMMU-132 has made a breakthrough, it is predictable that cancer cells will become resistant to this drug, so it is necessary to develop other targeted therapeutics.

EGFR is frequently and highly expressed in patients with TNBC and is associated with poor overall survival [26, 27]. EGFR-targeted therapies, including mAbs and small-molecule TKIs, have shown promising efficacy in a variety of tumor therapies, including non-small cell lung cancer, HER2-positive breast cancer, and head and neck cancer [28–30]. EGFR has been viewed as a promising therapeutic target. However, due to a poor response or the development of drug resistance, the therapeutic effect is not ideal in TNBC patients. Unlike mAbs or TKIs, which kill cancer cells only by inhibiting EGFR signaling, EGFR-targeting ADCs work mainly by binding to antigens on cancer cells, internalization into lysosomes and then releasing payloads to kill the cells. Previous studies have found that ADCs specifically targeting EGFR demonstrated promising therapeutic efficacy in some solid tumors, such as colorectal cancer and glioblastoma multiforme [31, 32]. We also reported a novel ADC LR004-VC-MMAE that showed therapeutic potential in esophageal squamous cell carcinoma. In this study, we further investigated its antitumor efficacies in EGFR-expressing TNBC. As shown in the results, in vitro, LR004-VC-MMAE significantly inhibited the proliferation, migration and invasion of EGFR-positive TNBC cells in a time- and dose-dependent manner. CSCs, which possess self-renewal and multilineage differentiation capacities, are considered to be an engine of tumor evolution [33, 34]. LR004-VC-MMAE also strongly inhibited the function of CSCs, as evidenced by the decreases in tumorsphere formation and reduced expression levels of CSC-related markers. Hence, LR004-VC-MMAE can not only kill tumor cells but also demonstrate a powerful killing ability against tumor stem cells. In vivo, LR004-VC-MMAE inhibited tumor growth and even achieved complete regression in TNBC xenograft models, which further indicated that LR004-VC-MMAE may represent a promising therapeutic candidate against EGFR-overexpressing TNBC.

It is well known that ADCs exert antitumor activity mainly through cytotoxicity derived from their payloads. LR004-VC-MMAE works against cancer cells mainly via the release of MMAE, which is a very potent antimitotic agent that inhibits cell division by blocking the polymerization of tubulin. However, activation of EGFR signaling is critical in cell proliferation, apoptosis, angiogenesis and other processes associated with cancer progression [35]. Although inhibiting EGFR signaling alone does not work in treating TNBC patients, we decided to investigate if it will play a role in LR004-VC-MMAE against TNBC. Surprisingly, we found that LR004-VC-MMAE not only specifically targeted EGFR but also inhibited EGFR expression and activation of the signaling pathway. As shown in the results, LR004-VC-MMAE promoted EGFR degradation and inhibited the phosphorylation of EGFR downstream signals including EGFR, MEK and ERK. It is worth noting that LR004-VC-MMAE also downregulated cancer stemness-related genes, such as Oct4, Sox2, KLF4 and EpCAM. This indicated that inhibiting the expression of tumor stemness-related genes may be involved in the antitumor mechanism of LR004-VC-MMAE.

However, how did LR004-VC-MMAE regulate stemness-related genes? Further investigation is required to determine whether this was achieved by suppressing EGFR signaling or by regulating other signaling pathways. In addition, drug resistance remains one of the biggest challenges in cancer therapy, which can lead to treatment failure and disease recurrence, as well as limits the effectiveness of most current treatments [36]. To address drug resistance and enhance the therapeutic effects, we plan to explore the antitumor effect of LR004-VC-MMAE on drug-resistant cell lines or combining it with some chemotherapy drugs in future research.

## Conclusions

In this study, we confirmed that an EGFR-targeted ADC (LR004-VC-MMAE) exerted excellent antitumor effects against EGFR-positive TNBC both in vivo and in vitro. Its mechanism of action was at least in part mediated by inhibiting the activation of EGFR signaling and the expression of cancer stemness marker genes. Although further mechanistic studies are needed, this study partly explained the reason why it demonstrated excellent antitumor activity and provided a new candidate drug targeting TNBC. It also proposed a potential avenue of therapeutic interventions for TNBC patients in the future.

## Supplementary Information

**Additional file 1: Fig. S1** Structure and characterization of LR004-VC-MMAE. **a** Chemical structure of LR004-VC-MMAE. **b** Conjugated drug distribution by HIC-HPLC analysis. The average DAR of LR004-VC-MMAE is 4.02. DAR drug-to-antibody ratio.

## Data Availability

The datasets used and/or analyzed in this article are available from the corresponding author upon reasonable request.

## Abbreviations

ADC: Antibody–drug conjugate
CSCs: Cancer stem cells
DAR: Drug-to-antibody ratio
DMEM: Dulbecco’s modified Eagle’s medium
EGFR: Epidermal growth factor receptor
ER: Estrogen receptor
GEO: Gene expression omnibus
HER2: Human epidermal growth factor receptor 2
HER3: Human epidermal growth factor receptor 3
HR: Hormone receptor
HIC: Hydrophobic interaction chromatography
LR004: Anti-EGFR antibody
mAb: Monoclonal antibody
MMAE: Monomethyl auristatin E
PI: Propidium iodide
PR: Progesterone receptor
RTK: Receptor tyrosine kinase
SD: Standard deviation
TKI: Tyrosine kinase inhibitor
TNBC: Triple-negative breast cancer
TV: Tumor volume
TROP-2: Trophoblast cell-surface antigen 2
VC: Valine-citrulline
