# Supplementary figures and images for: Excellent effects and possible mechanisms of action of a new antibody–drug conjugate against EGFR-positive triple-negative breast cancer
Source: Mil Med Res. 2021 Dec 9;8:63. doi: 10.1186/s40779-021-00358-9 (PMC8656065; doi:10.1186/s40779-021-00358-9)

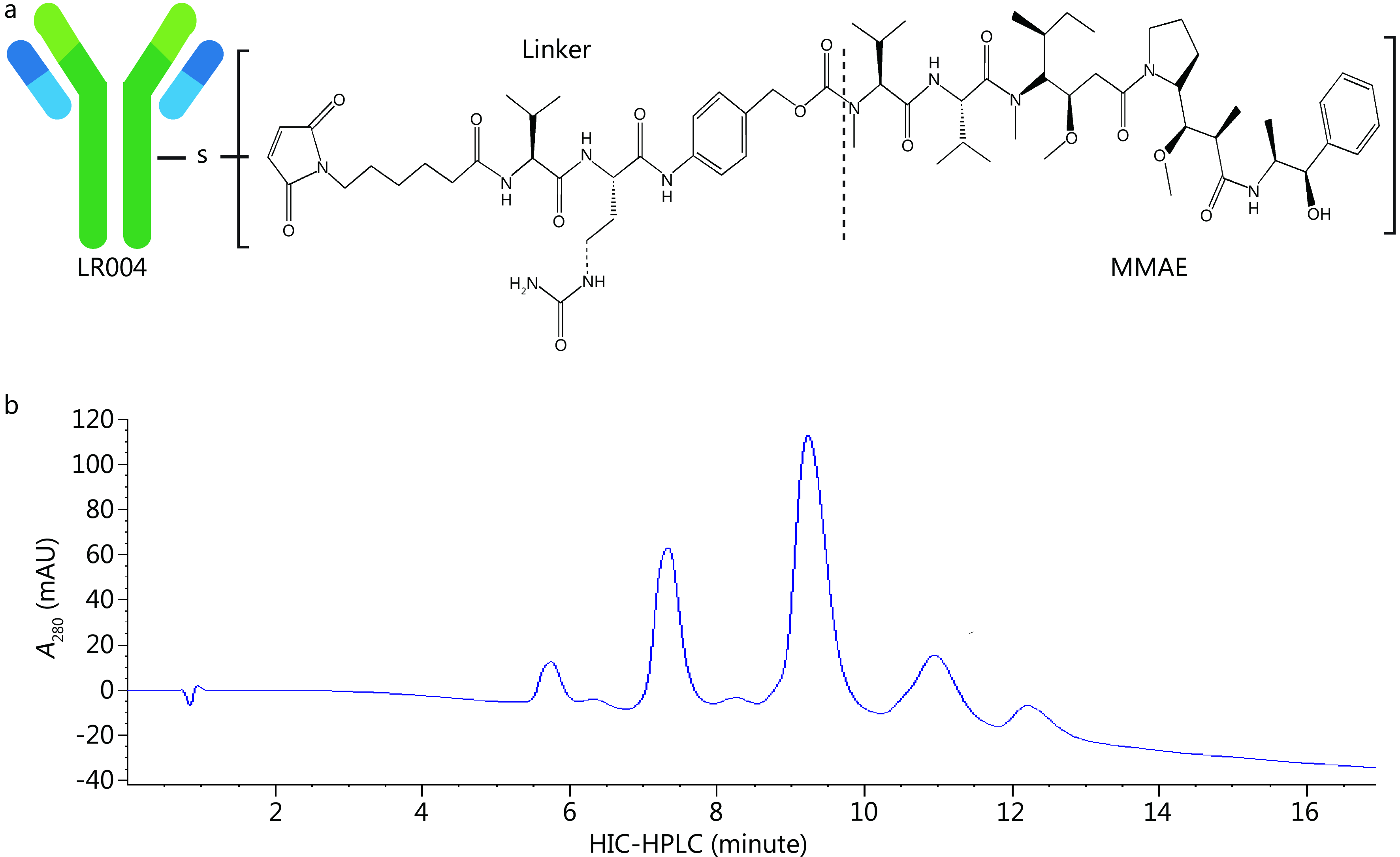

Supplement: Supplementary file 1 — Additional file 1: Fig. S1 Structure and characterization of LR004-VC-MMAE. a Chemical structure of LR004-VC-MMAE. b Conjugated drug distribution by HIC-HPLC analysis. The average DAR of LR004-VC-MMAE is 4.02. DAR drug-to-antibody ratio. [file 40779_2021_358_MOESM1_ESM.tif]
